# A European regulatory pathway for Tidepool loop following clearance in the United States?

**DOI:** 10.1111/dme.15246

**Published:** 2023-11-23

**Authors:** Laura Downey, Shane O’Donnell, Tom Melvin, Muireann Quigley

**Affiliations:** 1 Birmingham Law School, University of Birmingham, Birmingham, UK; 2 College of Business, University College Dublin, Dublin, Ireland; 3 School of Medicine, Trinity College, University of Dublin, Dublin, Ireland

**Keywords:** open-source automated insulin delivery systems, medical devices regulation, type 1 diabetes, user-led innovation

## Abstract

The recent clearance by the United States Food and Drug Administration of Tidepool Loop sets an important precedent within the medical device landscape. For the first time, an automated insulin delivery mobile application—based on an algorithm initially designed and developed by users —has been recognised as safe and effective by a regulatory body. The aim of this paper is twofold: firstly, we map out the regulatory pathways and processes that were navigated by Tidepool, the non-profit behind Tidepool Loop, in order to make this landmark moment possible. Secondly, we set out potential approvals processes in the European Union and United Kingdom with a view to examining the challenges to obtaining regulatory clearance for Tidepool Loop in these jurisdictions. In so doing, we highlight the significant differences, not only between the United States and European systems but also between the European Union and Great Britain systems. We conclude by arguing that the complexity encountered when seeking to introduce an innovative solution in different regulatory systems has the potential to act as a disincentive to open source developers from seeking regulatory approvals for such technologies in the future.

## 1 Introduction

In January 2023, Tidepool Loop became the first open source automated insulin delivery (OS AID) “mobile application with algorithm technology”^1^ to gain regulatory clearance by the United States Food and Drug Administration (US FDA). This is an important milestone in the history of the #WeAreNotWaiting movement and the medical devices landscape more generally. It not only represents the culmination of more than five years of effort by the diabetes non-profit Tidepool but also the countless contributions of people with diabetes (PwDs) from all over the world that made an open source approach to automated insulin delivery possible. In this paper, we look at the regulatory pathways and processes that were navigated in order to make this landmark moment possible. We then compare this to potential approvals processes in the European Union (EU) and United Kingdom (UK). To begin, however, in order to place Tidepool Loop in context, we give a short history of the #WeAreNotWaiting movement and the development of the OS AID algorithms which gave rise to it.

## 2 THE #WeAreNotWaiting MOVEMENT, TIDEPOOL (LOOP) AND REGULATORY CLEARANCE

In the early 2010s, tech savvy PwDs, frustrated with the slow pace of medical innovation in diabetes care, started to create new tools and systems and shared them for free via open-source platforms in order to help others to make better use of their devices and data. Rallying under the banner #WeAreNotWaiting, this movement has developed a range of innovations that are significantly more advanced than those available commercially to PwDs.^2^ From a regulatory perspective, the most significant of these are what have come to be known as open source automated insulin delivery (OS AID) systems. In these systems, treatment decisions are delegated to an algorithm that automates the process of insulin delivery based on a feedback-loop between a continuous glucose monitor (CGM) and insulin pump.^3^ One of the most commonly used versions of OS AID is “Loop”, an iOS app (which needs to be “built” by users themselves) that connects to an insulin pump and CGM using Bluetooth LE. Like other OS AID (Open APS, AndroidAPS, FreeAPS X), the basic implementation of Loop functions as a hybrid closed loop system. More specifically, the Loop algorithm works by “predict[ing] future glucose by adding together the effect of carbohydrates on increasing glucose, insulin on decreasing glucose and then two forms of short-term adaptation dubbed glucose momentum and retrospective correction”.^4^ Based on these projections, the app makes continuous adjustments to insulin delivery in order to meet user-defined glucose targets. Loop is compatible with older generation Medtronic pump models and Omnipod Eros and Dash, as well as most types of CGMs.

It is estimated that Loop is used worldwide by thousands of individuals.^5^ Nevertheless, significant barriers remain to their uptake among people living with diabetes. These include (but are by no means limited to) the (in)accessibility of components, lack of IT knowledge and fears of losing the support of healthcare providers.^6^ Such barriers to the wider diffusion of the innovations within the #WeAreNotWaiting movement prompted some within the community to explore pathways towards regulatory approval, with the goal of increasing availability to those not in a position to build such a system themselves. As such, borne out of frustration with the slowness of device/solution development and the ring fencing of access to these solutions,^7^ Tidepool was founded in 2013 by Howard Look, a parent of a child with type 1 diabetes who was an active user of the Loop.^8^ Tidepool’s vision is to encourage diabetes device companies to recognise the value of a more open, interoperable approach to medical device development. An early success in this regard was persuading most of the leading diabetes device manufacturers to provide Tidepool with their data/device protocols to enable the development of an interoperable diabetes data management platform. Subsequently, Tidepool made the decision to begin work on the development of a modified version of the Loop algorithm for regulatory submission to the US FDA.

In 2018, Tidepool officially launched “Tidepool Loop”, a project “dedicated to delivering an officially supported, FDA regulated version of the Loop app, making it broadly available to download via the iOS App Store”.^8^ In January 2023, Tidepool announced it had obtained market clearance for the United States (US), although to date no official timeline has been given for official launch. A significant impediment to making the app available on the market has been the absence of a commercial vendor willing and/or able to seek clearance from the FDA for Alternate Controller Enabled (ACE) pump designation. As we will see later, this is a perquisite for participation in the Tidepool ecosystem. As such, in the absence of an ACE pump partner, the launch of Tidepool Loop on the US market will have to wait.

In keeping with the spirit of the #WeAreNotWaiting community, the code for the Tidepool Loop programme is available on Github for anyone to copy, modify, and share. However, any significant changes to the Tidepool Loop control algorithm would need clearance from the FDA before it can be implemented in any future versions.^9^ In particular, the Tidepool Loop documentation from the FDA notes that change to the intended purpose and/or change that could affect safety and/or effectiveness of the system would require a new pre-market submission.^10^

Notwithstanding the challenges, Tidepool's success in stewarding Tidepool Loop through the US FDA clearance process provides an opportunity to look at how this landmark moment was achieved. It also prompts us to ask whether, and via what processes, something similar could happen within the EU and the UK. Thus, in the following sections, we examine the regulatory processes that Tidepool navigated and compare them to what would be required for its approval in the EU and UK contexts.

## 3 Regulatory Approvals And Classifying Medical Devices

In the US, the FDA regulates medical devices under powers granted by the Federal Food, Drug and Cosmetic Act. Meanwhile, medical devices (which are not in vitro diagnostic devices) in the EU are governed by Regulation (EU) 2017/745 on Medical Devices (MDR). This recently replaced the previous medical devices Directives: Directive 90/385/EEC concerning active implantable medical devices (AIMDD) and Directive 93/42/EEC concerning medical devices (MDD). A Competent Authority within each EU member state is responsible for regulation and enforcement nationally, while Notified Bodies across Europe are responsible for the conformity assessment of devices to be placed on the market. Following Brexit, the UK now has a dual system of regulation with regards to medical devices. Northern Ireland (NI) falls under the jurisdiction of the EU Regulations via the NI interpretation of the UK's Medical Devices Regulations 2002, while Great Britain (GB) is governed by the England, Wales and Scotland (E+W+S) interpretation of the 2002 Regulations. The GB version of the UK's Regulations, until they are changed, in essence incorporates the provisions of the older EU Directives. In the future, devices approved by other international systems may be recognised by the UK, something which is currently under consideration.^11^

Placing a medical device on the market, on which-ever side of the Atlantic, comes with a complex set of regulatory requirements. The level of oversight and particular processes for approvals, for any particular device, largely depends on the classification of that device. The US, EU (including NI) and GB all have risk-based systems for classifying devices, but there are some differences that should be kept in mind which will influence potential applications by Tidepool Loop for EU and GB approvals.

### 3.1 Tidepool Loop and the US FDA 510(k) process

In the US, there are 3 device classifications:^12^

- Class I devices are deemed the lowest risk and manufacturers wishing to bring them to market need only self-declare regulatory compliance in the majority of cases.
- Class II devices are moderately risky and have to go through either a 510(k) premarket notification process or a “De Novo” process.
- Class III devices are the highest risk devices and must undergo the more onerous premarket approval process.

Tidepool Loop gained regulatory clearance as a class II device through the 510(k) premarket notification process.^13^ This refers to section 510(k) of the Food, Drug and Cosmetic Act which is the principal regulation governing medical devices in the US. This pathway requires that the device in question is substantially equivalent to another already on the market.

A device is taken to be substantially equivalent if it has the same intended use and *either* has the same technological characteristics as the “predicate device”—the device on which the claim of substantial equivalence is based—*or* it can be shown to be just as safe and effective as the predicate device and raises no further distinct questions of safety.^14^ Tidepool Loop was able to use the 510(k) pathway because a predicate device existed: the Tandem Diabetes Care's Control-IQ Technology.^15^ Tandem's technology is a mobile app-based interoperable automated glycaemic controller (iAGC) which had already gained regulatory authorisation via a different pathway: the De Novo premarket review pathway.^16^ The De Novo process is used where manufacturers of novel devices (for which there is no existing predicate device with which they are substantially equivalent) that would otherwise automatically be deemed class III devices believe they should be given a lower classification. Once authorization is given for the device through the De Novo pathway, it can be relied on as a predicate device for future 510(k) applications. Because Tidepool Loop was determined to have substantial equivalency to a pre-existing device, the 510(k) pathway to approval was opened up. Although the 510(k) pathway does not typically require clinical studies as part of their application, one of the conditions of the De Novo authorisation (a “special control”) for the Control IQ technology is for subsequent devices of this type to provide clinical evidence of the device’s safety and clinical effectiveness.^17^ Given this, and with FDA concurrence of the study design, Tidepool Loop submitted results from a prospective observational study to support the clinical performance and safety of the device.^18^

The clearance by the FDA of the Tidepool Loop app was facilitated, at least in part, by the fact that the FDA allows the component devices in an AID system (CGM, insulin pump, plus mobile application) to be assessed individually, rather than as a system as a whole. This negates the need to assess all other devices in the system whenever clearance for a new device or an update to an existing one is sought.^19^ There are, however, specific requirements regarding the interoperability of devices which can be used together as part of an AID system. For example, for ACE insulin pumps, “special controls” are applied with respect to the design and labelling of the device which includes requirements relating to their communication with other compatible devices in the AID system.^20^ The requirements relating to this do not include a requirement that a pump be capable of being interoperable with *any or all* potential component devices. As such, AID component devices currently on the market, including ACE pumps, have so far been cleared or authorised as interoperable only with specified compatible devices, of which Tidepool Loop is not one. This means, as noted earlier, that despite having regulatory clearance, there is currently no commercially available ACE pump that can be used as part of the Tidepool Loop ecosystem.

### 3.2 Tightened requirements under the European Union Medical Devices Regulation versus Great Britain's older classification rules

Classification and approvals in the EU on the other hand are different. For medical devices, such as Tidepool Loop, that are *not* in vitro diagnostic devices, the MDR continues the previous classifications going from low to high risk: class I, class IIa, class IIb and class III. This follows a cascading rules system, based on device function and risk. The rules are set out in Annex VIII of the EU MDR. The classification rules and guidance on their application clearly make a distinction between standalone software—software considered a medical device in and of itself—and software that drives or influences a device or is an accessory to a medical device.^21^ Different rules apply depending on these distinctions with the potential for different possible classifications. The key element in making these distinctions is ascertaining whether the software has its own medical purpose within the MDR's definition of “medical device” distinct from any other device it might influence or drive. It is made clear on Tidepool's website, and from the “indications of use” outlined in a letter from the FDA, that Tidepool Loop is intended to automate insulin delivery which would fall within one of the listed medical purposes in the MDR. However, it is also intended to be used with other devices—a CGM and insulin pump—to achieve that purpose.

Despite this reliance on other devices for ultimate use, it is likely that Tidepool Loop would count as a device in its own right. The definition of “medical device” in the MDR specifically states that devices can be intended to be used “alone or in combination” with other devices. On an ordinary reading this implies that devices can have a distinct medical purpose even when used together with, or are reliant upon, other devices. This further implies, absent any explicit guidance on the matter, that all interoperable devices in an AID system in the EU (and in GB which retains a similar definition), including the hardware devices, can be assessed and approved individually (much like in the US). Furthermore, guidance on the interpretation of the classification rules confirm that where software influences other devices, it will still be considered standalone software—or medical device software (MDSW) in the words of the guidance—so long as it retains its own medical purpose.^22^ If, therefore, Tidepool Loop, is considered to be a piece of standalone software, and thus a device in its own right, then the classification rules relating to “active devices” are the applicable ones.^23^

It is difficult for us to say with certainty as to the exact classification which would apply to Tidepool Loop. This is because the eventual classification is largely dependent on the intended purpose as described by Tidepool Loop, and this could be different between the EU and US. However, under the most probable Rule, it would likely be deemed to be a Class III device, depending on which part of the rules was deemed to be applicable. Tidepool Loop most likely falls within the scope of Rule 11 as it is “[s]oftware intended to provide information which is used to take decisions with diagnosis or therapeutic purposes”. This rule, however, contains a couple of potential classifications. Where a device carries a risk of “a serious deterioration of a person's state of health”, it will be Class IIb.^24^ However, where there is a risk of “death or an irreversible deterioration of a person's state of health”, it would be a Class III device.^24^ It is arguable that such MDSW, through its insulin dosage calculation and administration functions, carries a risk of death and, therefore, ought to be considered as a Class III device.

Rule 22 may also apply. This rule states that “active therapeutic devices with an integrated or incorporated diagnostic function which significantly determines the patient management by the device” are Class III devices.^25^ Although Tidepool Loop is software, and this rule appears to be framed with physical devices in mind, there is an implementing rule that notes that “software, which drives a device or influences the use of a device, shall fall within the same class as the device”.^26^ Indeed, in the classification guidance from the Medical Device Coordination Group, automated closed loop insulin delivery systems are specifically given as examples of devices which take the higher Class III classification.^27^ In any case, following either pathway will bring Tidepool Loop to Class III.

We should note that two comparator products – Diabeloop and CamAPS FX – which both gained a CE mark in the EU are class IIb devices.^28^ However, approvals for these were gained in 2018 and 2020, respectively.^29^ As such, their conformity assessments and CE marking were done under the requirements of the older MDD, before the new EU MDR was in force, and their continuing placement on the market is only allowable under so-called legacy certification.^29^ This permits devices which were conformity assessed and gained certification under the MDD to continue to be placed on the market during the transitional period between the two regimes.^30^ Arguably, therefore, once the transitional period ends, these will also be deemed to be class III devices under the EU MDR and, as such, will likely be up-classified and, therefore, need to meet the more stringent requirements this brings.

As is apparent from the discussion so far, the EU MDR changed the detail of the classification rules and as a result, some devices may fall under a different risk classification compared with the MDD. This has implications for GB, because, as noted earlier, the GB interpretation of the UK's Medical Device Regulations 2002 derives from the older Directives and the classification rules that it applies. The principal difference, in practical terms, is that Tidepool Loop would in all probability be assigned to Class IIb under these classification rules. Here Rule 9 of the MDD is the applicable one. This says that “[a]ll active devices intended to control or monitor the performance of active therapeutic devices in Class IIb, or intended directly to influence the performance of such devices are in Class IIb”. As a device which administers a “potentially hazardous” medicine,^31^ an insulin pump is a Class IIb device under the GB-relevant provisions of the 2002 Regulations. Accordingly, as Tidepool Loop in essence controls the performance of the insulin pump, it would also be a Class IIb device under the current GB regulations.

### 3.3 Conformity assessment: using device equivalence?

The routes to approval in the three jurisdictions discussed here are determined by the classification of the device. The main difference between the US and EU/GB approvals processes is the requirement for clinical evidence. In the US, class III devices subject to the pre-market approval pathway always require clinical studies, devices in the de novo pathway often require clinical studies, and devices subject to the 510(k) pathway occasionally submit clinical studies. With the 510(k) route the focus is on the determination of substantial equivalence to a predicate. In contrast, in the EU and GB a clinical evaluation is required for all devices required to undergo a conformity assessment by a Notified/Approved Body. Within the different risk classes, Class III devices are subject to a more rigorous assessment than lower risk devices. The clinical evidence requirements in the older Directive system, and still relevant to GB, have been changed under the EU MDR.

One important change relates to the ability to claim equivalence to another device. The EU MDR introduced tightened equivalency requirements in respect of the biological, technical and clinical criteria.^32^ Additionally, reliance on data from equivalent devices for high-risk devices in the EU now requires a contract with the equivalent device manufacturer. The contract must specify that access will be allowed to the full technical documentation of the claimed equivalent device.^33^ This was not needed under the MDD. As a result, it may be possible for Tidepool Loop to claim equivalence for the purpose of GB market entry, if they meet the requirements set out in the operative guidance.^34^ The evidentiary burden in relation to this may also be eased given that some comparator products, as already mentioned, have previously been approved in the EU under the MDD. However, in the EU, the equivalency process will be more challenging given the need for a contract under the MDR, something which might prove difficult for organisations such as Tidepool to obtain.

Having said this, in light of the recent MHRA consultation on the future of medical devices regulation, and the Government response, this is not assured.^35^ Many of the changes brought about by the EU MDR look set to be emulated in forthcoming Regulations in the UK/GB. This includes tighter requirements on equivalency going beyond those set by the EU MDR to “entire equivalence”.^36^ The Government's rationale for this, despite acknowledging the potential effect on innovation, is to avoid “product creep” where new devices placed incrementally on the market can end up far from original predicate devices.^36^ Nevertheless, for the time being, any application will be subject to the current system. This might work in favour of Tidepool Loop in terms of regulatory burden, but ultimately it is not clear how easy navigating a system in transition, such as the UK/GB, would be. In any event, the clinical investigation conducted to support the 510(k) clearance in the US should constitute clinical data for the purpose of conformity assessment to both EU and GB regulations (see Table 1 above).

What neither the EU nor UK regulatory systems have is any technology specific controls similar to the “special controls” applied by the US FDA and outlined earlier in relation to device interoperability. While the essential requirements laid down in the EU MDR and the UK’s 2002 Regulations are specific to the device categories they apply to, they are general in the sense that they apply to *all* devices in those categories. Having said this, however, manufacturers can demonstrate compliance with recognised international standards in order to show that their device meets the essential requirements, something which can be technology specific.

## 4 Concluding Thoughts

The clearance of Tidepool Loop is an excellent example of how user-driven innovation has the potential to change the existing medical landscape. From a regulatory perspective, it is also an excellent example of the complexity that can be encountered when seeking to introduce an innovative solution in different regulatory systems. In an ideal regulatory system, the evidence requirements would not differ significantly across different jurisdictions. Yet, as we have seen in this paper, there can be significant differences, not only between the US and European systems but also between the EU and GB systems. This, amongst other things such as not being able to gain a contract with an equivalent device manufacturer, has the potential to act as a disincentive to open source developers from seeking regulatory approvals for such technologies.

The US FDA, as a single-agency model of regulation, can adapt existing pathways to better suit disruptive technologies. For instance, as noted earlier, Tidepool Loop can introduce new pumps into its ecosystem subject to a pre-specified process agreed with FDA as part of the 510(k) process. This may be challenging under the EU or GB rules where the rules and guidance place an emphasis on changes to the design or intended purpose.^37^ The US FDA can also support technology developers with questions relating to device development by means of a “Q-submission” meeting, in which developers can meet with FDA staff to discuss a device or application and get feedback on these.^38^ This is also not available within the EU, although “structured dialogue” with a Notified Body is now encouraged by the Medical Device Coordination Group.^39^

Regulatory approval allows market access. However, it does not guarantee user access. For many health systems, a separate assessment is required to determine if the device should be made available on, or reimbursed by, national healthcare systems. In this paper, we explored the regulatory and market access considerations in relation to Tidepool Loop; the first open source automated insulin delivery system to gain regulatory approval in any jurisdiction. We did not, however, consider health technology availability or reimbursement in this case or more generally speaking. While there is a lot of forthcoming change in this area both in the UK^40^ and in the EU,^41^ automated insulin delivery provision from within the healthcare system is likely to remain either unavailable or unaffordable for many people with diabetes in Europe, the UK, and US for some time to come.

Meanwhile, industry continues to lag behind when it comes to embracing changes to device development that are not only demanded of them by patients, but, as the #WeAreNotWaiting movement has demonstrated, are also eminently achievable in practice. For example, fully closed loop systems have yet to have a commercial debut, despite significant advances in this area made by OS AID innovators. Furthermore, full interoperability between different diabetes devices has not been widely or adequately pursued by device manufactures,^4^ as reflected in Tidepool's lack of success thus far in securing a pump partner to support the launch of Tidepool Loop onto the US market. Likewise, commercial manufacturers have not adopted core values, such as full transparency in how algorithms operate and unconditional access to device data for end-users, something which is at the heart of the OS AID community.^4^ Given this, OS solutions are likely to continue to be seen as an option for PwDs who feel they cannot wait for commercial and regulatory actors to catch-up.

## Figures and Tables

**Table 1:** Comparison of regulatory pathways and requirements in the United States, Europe and Great Britain for MDSW such as Tidepool Loop.

	United States	European Union	Great Britain
**Legislation**	Food, Drug, & Cosmetic Act	Regulation (EU) 2017/745 on Medical Devices	Medical Devices Regulations 2002 (as amended)
**Regulatory pathway or applicable rules**	501(k)	Rule 11 (or 22)	Rule 9
**Device classification**	Class II	Class III	Class IIb
**Clinical evidence required**	May submit, not strictly required	Yes	Yes
**Can claim equivalence with other devices**	Yes, evidence of ‘substantial equivalence’ required. In practice, this may mean clinical evidence as it can be difficult to demonstrate equivalence without it	Yes, evidence of ‘equivalence’ required, overall tighter requirements as per MDR Article 61(5). Must have a contract with an equivalent manufacturer allowing full access to technical documentation	Yes, evidence of ‘equivalence’ required as per MEDDEV 2.7/1, revision 4 guidance. Possible tightening of rules in the future to only allow ‘entire equivalence’

## Data Availability

Data sharing not applicable to this article as no datasets were generated or analysed during the current study.

## Novelty Statement

- This study is the first to consider the implications in a European context of the recent clearance of Tidepool Loop by the United States Food and Drug Administration.
- Regulations in the United States are compared with those of the European Union and United Kingdom with a view to mapping out the pathway for user-led innovations like Tidepool Loop to be approved in Europe in the future.
- The article highlights how the complexities of navigating both the European Union and United Kingdom regulatory regimes might dissuade open source developers from seeking regulatory approval for their innovations.
